# New Appliances and Things Medical

**Published:** 1905-07-08

**Authors:** 


### NEW APPLIANCES AND THINGS MEDICAL.

[We shall be glad to receive, at our Office, 28 & 29 Southampton Street, Strand, London, W.C., from the manufacturers, specimens of all new preparations and appliances which may be brought out from time to time.]

### NURSING OXO.

(Liebig's Extract of Meat Company. 4 Lloyds Avenue, London, E.C.)

A copious literature has arisen concerning beef-tea and the many liquid preparations which can be made of meat and are rapidly available for the invalid. Drastic criticism has been passed upon those preparations of meat which contain, both as sold and as consumed by the invalid, only so called extractives, which, in that these substances and gelatine possess in the strict sense of the word no food value, is justified. It must, however, be admitted that these so-called food values are often arrived at by chemico-physical methods, which, from the physiological point of view, are very often exceedingly arbitrary. From the point of view, also, of the clinician, a stimulant pure and simple is often of as great value as a food, since by its use energy, otherwise unavailable for the patient, can be unlocked. Few patients die of actual starvation, but many because they cannot convert their potential into kinetic energy. If a stimulant, whether it be beef-tea, coffee, tea, or gelatine enables them to do this, it must possess a distinct value. Hence, in the opinion of the writer, the mere fact that a meat preparation has been weighed in the calorimetric balance and found wanting should not lead physicians to regard it as necessarily useless. The preparation before us is a modification of one of the earliest preparations of this kind ever put on the market, viz., Liebig's extract of meat. We have not made an analysis of this substance, but we are informed that it contains not only the extract of meat, but also peptone and "fibrin"; by the latter, we assume, is meant soluble proteid. When made according to the directions the flavour is excellent, and although there are numerous preparations of this kind already on the market, Nursing Oxo will, we have no doubt, hold its own and find an extensive sphere of usefulness.

